# Alcohol consumption and cause-specific mortality in Cuba: prospective study of 120 623 adults

**DOI:** 10.1016/j.eclinm.2020.100692

**Published:** 2021-03-17

**Authors:** Nurys B. Armas Rojas, Ben Lacey, Daniel Martin Simadibrata, Stephanie Ross, Patricia Varona-Pérez, Julie Ann Burrett, Marcy Calderón Martínez, Elba Lorenzo-Vázquez, Sonia Bess Constantén, Blake Thomson, Paul Sherliker, José Manuel Morales Rigau, Jennifer Carter, M. Sofia Massa, Osvaldo Jesús Hernández López, Nazrul Islam, Miguel Ángel Martínez Morales, Ismell Alonso Alomá, Fernando Achiong Estupiñan, Mayda Díaz González, Noel Rosquete Muñoz, Marelis Cendra Asencio, Jonathan Emberson, Richard Peto, Sarah Lewington

**Affiliations:** a National Institute of Cardiology and Cardiovascular Surgery, Havana, Cuba; b Nuffield Department of Population Health (NDPH), University of Oxford, UK; c Faculty of Medicine, Universitas Indonesia, Jakarta, Indonesia; d Institute of Hygiene, Epidemiology and Microbiology, Ministry of Public Health, Havana, Cuba; e Cuban Commission Against Smoking, Ministry of Public Health, Havana, Cuba; f Directorate of Medical Records and Health Statistics, Ministry of Public Health, Havana, Cuba; g George Institute for Global Health, University of Oxford, UK; h MRC Population Health Research Unit, NDPH, University of Oxford, UK; i Provincial Center of Hygiene, Epidemiology and Microbiology, Matanzas, Cuba; j Municipal Center of Hygiene, Epidemiology and Microbiology, Jagüey Grande, Matanzas, Cuba; k Municipal Center of Hygiene, Epidemiology and Microbiology, Colón, Matanzas, Cuba; l Municipal Center of Hygiene, Epidemiology and Microbiology, Camagüey, Cuba; m UKM Medical Molecular Biology Institute (UMBI), Universiti Kebangsaan Malaysia, Kuala Lumpur, Malaysia

**Keywords:** Cuba, Alcohol, Mortality, Prospective study

## Abstract

**Background:**

The associations of cause-specific mortality with alcohol consumption have been studied mainly in higher-income countries. We relate alcohol consumption to mortality in Cuba.

**Methods:**

In 1996-2002, 146 556 adults were recruited into a prospective study from the general population in five areas of Cuba. Participants were interviewed, measured and followed up by electronic linkage to national death registries until January 1, 2017. After excluding all with missing data or chronic disease at recruitment, Cox regression (adjusted for age, sex, province, education, and smoking) was used to relate mortality rate ratios (RRs) at ages 35–79 years to alcohol consumption. RRs were corrected for long-term variability in alcohol consumption using repeat measures among 20 593 participants resurveyed in 2006-08.

**Findings:**

After exclusions, there were 120 623 participants aged 35-79 years (mean age 52 [SD 12]; 67 694 [56%] women). At recruitment, 22 670 (43%) men and 9490 (14%) women were current alcohol drinkers, with 15 433 (29%) men and 3054 (5%) women drinking at least weekly; most alcohol consumption was from rum. All-cause mortality was positively and continuously associated with weekly alcohol consumption: each additional 35cl bottle of rum per week (110g of pure alcohol) was associated with ∼10% higher risk of all-cause mortality (RR 1.08 [95%CI 1.05-1.11]). The major causes of excess mortality in weekly drinkers were cancer, vascular disease, and external causes. Non-drinkers had ∼10% higher risk (RR 1.11 [1.09-1.14]) of all-cause mortality than those in the lowest category of weekly alcohol consumption (<1 bottle/week), but this association was almost completely attenuated on exclusion of early follow-up.

**Interpretation:**

In this large prospective study in Cuba, weekly alcohol consumption was continuously related to premature mortality. Reverse causality is likely to account for much of the apparent excess risk among non-drinkers. The findings support limits to alcohol consumption that are lower than present recommendations in Cuba.

**Funding:**

Medical Research Council, British Heart Foundation, Cancer Research UK, CDC Foundation (with support from Amgen)

**Research in context**

**Evidence before this study**

A recent meta-analysis of prospective studies, with over 0.5 million current drinkers, described inverse associations of alcohol consumption with myocardial infarction but positive associations with other cardiovascular diseases. Overall, there was no evidence of a protective effect of alcohol consumption on all-cause mortality at any level of alcohol consumption; the threshold for the lowest risk of all-cause mortality was about 100g/week (equivalent to 12.5 UK units of alcohol per week). The studies contributing to this meta-analysis were from 19 high-income countries; none were from Cuba or Latin America.

**Added value of this study**

This is the first large-scale study on the relation between alcohol consumption and premature mortality in Latin America. In Cuban adults, higher alcohol consumption among weekly drinkers was positively associated with all-cause mortality throughout the range examined: each additional 35cl bottle of rum (∼14 UK units) per week was associated with about 10% higher risk of all-cause mortality. There was no evidence of excess mortality among occasional drinkers (ie, less than weekly drinkers). Non-drinkers had ∼10% higher risk of all-cause mortality than those drinking <1 bottle of rum per week, but the analyses indicated that reverse causality is likely to account for much of this apparent excess risk.

**Implications of all the available evidence**

This prospective study provides reliable evidence on the relation between alcohol consumption and cause-specific mortality in Cuba. The findings support limits to alcohol consumption that are lower than present recommendations in Cuba, and reinforce the importance of alcohol to non-communicable disease prevention in the Latin American region.

Alt-text: Unlabelled box

## 1 Introduction

High alcohol consumption has been strongly associated with several causes of death, including liver cirrhosis, pancreatitis, several cancers (upper aerodigestive, liver, breast, and bowel), and external causes [1,2,3]. Some studies have also reported moderate alcohol consumption to be associated with lower risk of death from other causes, notably vascular disease, such that moderate drinking in some populations has been associated with lower risk of all-cause mortality [[4], [5], [6]].

In 2018, a meta-analysis of prospective studies from 19 high-income countries, with over 0.5 million current drinkers, described inverse associations with death from myocardial infarction but positive associations with other vascular causes. Overall, the threshold for the lowest risk of all-cause mortality in this study was about 100g/week (equivalent to 12.5 UK units of alcohol per week) [1].

The 2015 UN Sustainable Development Goals include the prevention and treatment of harmful alcohol use as a global goal [7]. Yet, uncertainty remains about the thresholds for alcohol consumption associated with the lowest risk of mortality in low-income or middle-income countries, where the types of alcohol and patterns of consumption may be very different from those in high-income countries. In particular, there have been no large-scale prospective studies in Cuba or Latin America of the associations between alcohol and cause-specific mortality with which to inform evidence-based health policy on alcohol in the region.

## 2 Methods

### 2.1 Study design and participants

Details of the study design and survey methods have been reported previously [8]. Briefly, adults were recruited from the general population of five areas in Cuba (the capital city, Havana (Ciudad de La Habana), and four regional provinces: Pinar del Río, La Habana, Matanzas, and Camagüey) between January 1, 1996, and November 24, 2002. Family medical practices were randomly selected within each province (215 clinics were approached, and none refused to participate) and clinical staff (mainly physicians) sought to recruit all adults aged 30 years and older living in the clinic's catchment area (74% of adults agreed to participate). During household visits, clinical staff recorded information on participants’ age, sex, education, occupation, alcohol consumption, smoking habits, medical history, and current medication use; measurements of height, weight and blood pressure were subsequently taken at the local clinic (the original questionnaire in Spanish and the English translation are available in the appendix pp 2–3).

Participants who drank alcohol were asked to report their frequency of drinking (in days per week) for rum (or other spirits) and for beer, and the average number of measures (‘lineas’) of rum, or standard bottles (35cl) of beer, that they would normally consume on such days. The reporting of alcohol consumption was limited to rum and beer only because wine or other types of alcoholic drink were rare in Cuba at the time of the baseline survey. Since the main source of alcohol was rum, total alcohol consumption (frequency multiplied by amount, with beer taken as 0.125 times rum in strength) was described in units of bottles of rum (35cl of 40% pure alcohol by volume, equivalent to 110g of pure alcohol or ∼14 [UK] units of alcohol) per week.

Participants were categorised by alcohol consumption into non-drinkers, occasional drinkers (those who reported their normal quantity of alcohol consumption on an occasion that they drank, but did not drink weekly) and regular drinkers (who reported drinking at least weekly, and were further divided into those who drank alcohol equivalent to <1 bottle of rum per week, 1-<3 bottles per week or ≥3 bottles per week). As it was not possible to disaggregate non-drinkers into never and ex-drinkers (and as such address the potential for reverse causality from ex-drinkers who stopped due to ill-health), the analyses focus particularly on current drinkers.

To assess any changes in alcohol consumption during the follow-up period in each category of baseline-reported consumption, and to correct for regression dilution bias that may result from using baseline alcohol consumption only to assess the associations with disease risks, participants in some provinces were resurveyed in 2006–08 (on average 8.6 years after recruitment; 20 593 adults had complete information on alcohol consumption) with the same procedures as at recruitment. Ethics was approved by the National Institute of Cardiology and Cardiac Surgery, Havana (ref 0404134); all participants provided written informed consent.

### 2.2 Mortality follow-up

Study participants were followed up until January 1, 2017. Follow-up was censored at the date of death, the end of the risk period under consideration, the date of loss to follow-up, or at the end of the follow-up period. Deaths were identified through linkage by national identification number to the Cuban Public Health Ministry's national mortality records. In Cuba, almost all deaths are certified by a doctor, with the underlying and contributing causes of death coded according to standard WHO recommendations. The underlying causes of death were coded using the International Classification of Diseases ninth and tenth editions (ICD-9 and ICD-10). We used ICD-9 codes for deaths between 1996 and 2000, and ICD-10 codes for deaths from 2001 onwards (appendix p 4). Study participants who emigrated were considered lost to follow-up; however, emigration in Cuba is low [9].

### 2.3 Statistical analysis

Participants with incomplete data on alcohol or covariates were excluded from analyses, as were those with prior history of disease that may affect drinking patterns (ie, myocardial infarction, angina, stroke, chronic obstructive pulmonary disease, liver cirrhosis, chronic kidney disease, peptic ulcer, and cancer) to limit the effects of reverse causality, and those with no follow-up at ages 35–79 years (appendix p 5).

Cox regression was used to relate mortality rate ratios (RRs) to categories of alcohol consumption, with regular drinkers that drank <1 bottle per week as the reference category. Confidence intervals (CIs) were calculated using the variance of the log risk, which appropriately attributes variance to all groups (including the reference group with RR of 1.0), and as such allows CIs to be used to compare risks in any two groups [10,11]. Analyses in these models were stratified by sex and province (5 provinces) to allow for non-proportional hazards with respect to these covariates, and adjusted for age (in five-year groups of age at risk, 35–79), education completed (5 groups: less than primary, primary, secondary, high school [including technical school], university) and smoking (6 groups: never, ex-smoker, current smoker of less than 20 cigarettes per day, 20 cigarettes per day, more than 20 cigarettes per day, cigar only smoker). For regular drinkers, RRs were plotted against mean alcohol intake (35cl bottles of rum per week) at resurvey within each baseline-defined alcohol group, to give the associations with long-term average (or ‘usual’) intake [12]. The slope of the regression line was calculated and expressed as RR per 35cl bottles of rum per week.

Subgroup analyses were by age at risk (2 groups: 35–69, 70–79 years), by sex, and by smoking (2 groups: never, ever). RRs for these subgroup analyses were controlled, where appropriate, for the same set of covariates as the overall analyses; analyses by age at risk were further adjusted by five-year groups of age at risk within age groups 35–69 and 70–79 years, and analyses by smoking were further adjusted for amount smoked within ever smokers.

Sensitivity analyses were conducted to further adjust for potential confounders, including diabetes, occupation (8 groups: none, manager, professional, technician, service worker, agricultural, industrial, military), marital status (currently married, not currently married) and body-mass index (BMI). In addition, to examine whether the RRs varied with time, the adjusted RRs were estimated separately for each 5-year period of follow-up.We estimated the absolute excess risk of premature death (ie, at aged 35–69 years) at different levels of drinking by multiplying the RRs by a common factor so that the inverse-variance weighted average of the new standardised RRs matched the Cuban national mortality rate at ages 35–69 years in 2010 [13]. We used SAS (version 9.4) for statistical analyses, and graphs were plotted with R (version 3.6.2).

### 2.4 Role of the funding source

The funders of the study had no role in the study design, data collection, data analysis, data interpretation, or writing of the report. The corresponding author had full access to all the data and had final responsibility for the decision to submit this manuscript for publication.

## 3 Results

In total, 146 556 adults were recruited into the study. Of these, 639 were excluded because of missing information on alcohol or other covariates, 19 911 were excluded because they had prior chronic disease, and 7489 had no follow-up at ages 35 to 79 years, leaving 120 623 participants (exclusion criteria were not mutually exclusive; appendix p 5).

Among participants included in the main analysis, mean age was 52 years (SD 12), 67 694 (56%) were women, mean body-mass index was 24.3 kg/m^2^ (SD 4.1), mean systolic blood pressure was 124 mmHg (SD 15), and 6272 (5%) were diabetic (Table 1). A higher proportion of men reported being current tobacco smokers (ie, cigarettes or cigars) as compared to women (50% versus 27%, respectively). Alcohol consumption at recruitment was strongly correlated with smoking and lower educational level, but less strongly related with other factors.

**Table 1:** Baseline characteristics of the 120 623 participants included in the mortality analyses, by level of alcohol consumption.

	Non-drinkers	Occasional drinkers	Regular drinkers (35cl bottles of rum per week)			Total
			<1	1-<3	≥3	
Number of men	30 259	7237	4587	5904	4942	52 929
Number of women	58 204	6435	1724	882	449	67 694
Rum consumption, bottles per week	-	-	0.4 (0.3)	1.3 (0.7)	5.1 (3.9)	-
Beer consumption, units of 110g alcohol per week*	-	-	0.2 (0.2)	0.5 (0.6)	1.8 (2.7)	-
Total alcohol consumption, units of 110g alcohol per week*	-	-	0.5 (0.3)	1.8 (0.6)	6.9 (4.6)	-
Age, years	53 (12)	50 (11)	50 (11)	49 (10)	49 (10)	52 (12)
No formal education beyond primary school	29 370 (33%)	3609 (26%)	1773 (28%)	1920 (28%)	1709 (32%)	38 841 (32%)
Current smoker (in men)	12 497 (41%)	3684 (51%)	2605 (57%)	3950 (67%)	3534 (72%)	26 253 (50%)
Current smoker (in women)	14 435 (25%)	2104 (33%)	802 (47%)	534 (61%)	301 (67%)	18 142 (27%)
BMI, kg/m^2^	24.3 (4.2)	24.2 (3.8)	24.1 (3.8)	24.1 (3.6)	24.0 (3.9)	24.3 (4.1)
Systolic blood pressure, mm Hg	124 (16)	124 (13)	125 (14)	126 (14)	125 (14)	124 (15)
Diastolic blood pressure, mm Hg	80 (10)	79 (9)	80 (9)	81 (9)	81 (10)	80 (10)
Diabetes	4689 (5%)	629 (5%)	265 (4%)	305 (5%)	280 (5%)	6272 (5%)
Taking anti-hypertensives	13 623 (15%)	2256 (17%)	1035 (16%)	1004 (15%)	890 (17%)	18 817 (16%)

Data are n (%) or mean (SD). Results are standardised to the age and sex of the 120 623 participants (except mean alcohol consumption). Participants with no follow-up at ages 35-79 years, those with pre-existing chronic disease at baseline, and those with incomplete information on alcohol or covariates were excluded. Non-drinkers includes never drinkers and former drinkers; occasional drinkers are those that reported drinking less than weekly; and regular drinkers drank at least weekly (stratified by bottles of rum consumed per week, or equivalent total alcohol from any source).

⁎ Mean consumption is expressed in units of 110g of pure alcohol (the alcohol content of one 35cl [40% proof] bottle of rum).

**Fig. 1:**
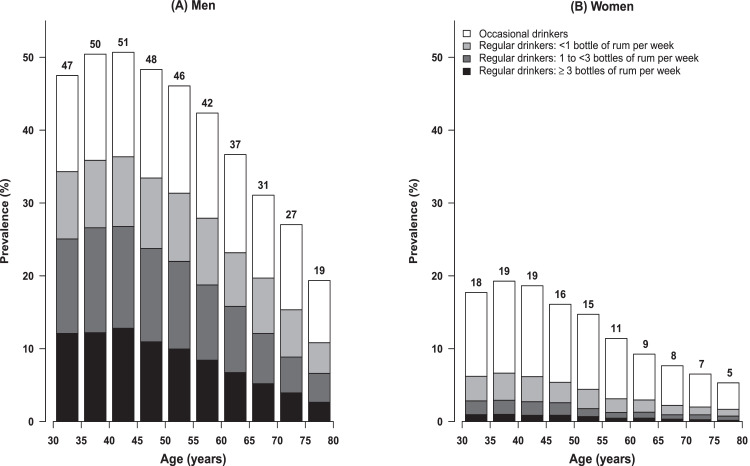
**Prevalence of alcohol consumption at baseline, by age and sex.** Proportion of participants at baseline (1996–2002) who were occasional (ie, less than weekly) drinkers and regular (ie, at least weekly) drinkers, with the latter subdivided into categories of weekly alcohol consumption (expressed as equivalent rum intake). One 35cl bottle of rum has approximately 110g of pure alcohol, or ∼14 UK units of alcohol. Exclusions and conventions as in Table 1.

At recruitment, 15 433 (29%) men and 3054 (5%) women were regular drinkers (drinking at least weekly), and 7237 (14%) men and 6435 (10%) women were occasional drinkers (drinking less than weekly) (Table 1). In both men and women, alcohol consumption at recruitment varied by age, with the prevalence of regular and occasional drinking decreasing with age (Fig. 1). Most regular drinkers drank rum only, or rum in combination with beer (appendix p 6); among regular drinkers, alcohol consumption per capita from beer was about a third of that from rum (Table 1).

Among resurveyed participants who reported drinking regularly at baseline, 1580 (74% of 2147) reported continuing to drink regularly at resurvey (mean 8.6 years later) (appendix p 7). Almost all (17 936 [98%]) of the 18 298 non-drinkers at baseline that were resurveyed remained non-drinkers, whereas few occasional drinkers at baseline (3 [2%] of 148) remained so at resurvey, with over half becoming regular drinkers. Mean alcohol consumption at resurvey among regular drinkers at baseline who drank <1 bottle of rum per week, 1-<3 bottles per week or ≥3 bottles per week, was 0.5 bottles, 1.5 bottles and 4.2 bottles, respectively (appendix p 7).

During 2.1 million person-years of follow-up at ages 35 to 79 years (mean 17 [SD 4] years per person), there were 13 556 deaths (6575 in women and 6981 in men; mean age at death 68 [SD 9] years); 232 participants were lost to follow-up. 5203 deaths were vascular, 4260 were cancer, 1282 respiratory disease, 187 liver cirrhosis, 1842 other medical causes, and 736 external causes.

**Fig. 2:**
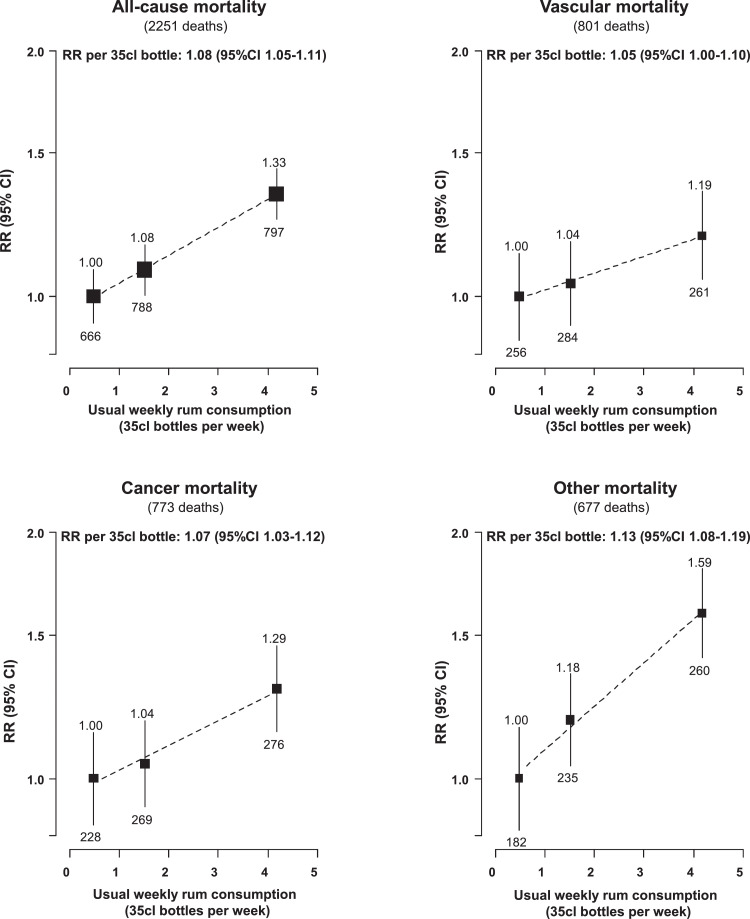
**Usual alcohol consumption among regular drinkers vs all-cause, vascular, cancer and other mortality at ages 35–79 years in Cuba.** Rate ratio (RR) adjusted for age, sex, education, province, and smoking. Analyses restricted to regular (ie, at least weekly) drinkers; conventions and other exclusions as in Table 1. For each alcohol category, area of the square is inversely proportional to the variance of the category−specific log risk, which also determines the confidence interval (CI).

Among regular drinkers, weekly alcohol consumption was positively associated with all-cause mortality after controlling for age, sex, province, education and smoking (Table 2, Fig. 2; smoking was found to be a particularly strong confounder of the association [see appendix p 8]). The association was approximately log-linear, with each additional 35cl bottle of rum per week (equivalent to 110g of pure alcohol) associated with about 10% higher risk of all-cause mortality (RR [95% CI] 1.08 [1.05-1.11]; 1.05 [1.03-1.07] without correction for regression dilution). Additionally, the comparison of RRs over the follow-up period indicated that there was no evidence of the proportional hazards assumption being violated (heterogeneity, 4 groups: χ^2^=0.47 [*p*=0.92]; appendix p 9).

**Table 2:** Alcohol consumption vs all-cause mortality in Cuba, by age, sex, and smoking.

	Non-drinkers		Occasional drinkers		Regular drinkers (35cl bottles of rum per week)						RR per 35cl bottle of rum per week*	
					<1		1-<3		≥3			
	Deaths	RR(95%CI)	Deaths	RR(95%CI)	Deaths	RR(95%CI)	Deaths	RR(95%CI)	Deaths	RR(95%CI)	Deaths	RR(95%CI)
Age-at-risk (years)												
35-69	4841	1.10 (1.06-1.14)	803	1.03 (0.96-1.11)	391	1.00 (0.90-1.11)	509	1.10 (1.01-1.20)	542	1.37 (1.26-1.50)	1442	1.09 (1.05-1.13)
70-79	5056	1.13 (1.09-1.18)	605	1.05 (0.97-1.14)	275	1.00 (0.89-1.13)	279	1.04 (0.92-1.17)	255	1.25 (1.10-1.42)	809	1.06 (1.02-1.11)
Sex												
Men	4049	1.06 (1.03-1.09)	901	1.02 (0.96-1.09)	554	1.00 (0.92-1.09)	723	1.05 (0.98-1.13)	754	1.30 (1.21-1.40)	2031	1.08 (1.05-1.11)
Women	5848	1.35 (1.31-1.39)	507	1.20 (1.09-1.31)	112	1.00 (0.83-1.20)	65	1.11 (0.87-1.42)	43	1.33 (0.98-1.79)	220	1.06 (0.98-1.14)
Smoking												
Ever	4460	1.08 (1.04-1.11)	895	1.00 (0.94-1.07)	498	1.00 (0.92-1.09)	654	1.08 (1.00-1.16)	687	1.33 (1.23-1.43)	1839	1.08 (1.05-1.11)
Never	5437	1.19 (1.16-1.23)	513	1.13 (1.04-1.24)	168	1.00 (0.86-1.16)	134	1.05 (0.88-1.24)	110	1.31 (1.08-1.58)	412	1.09 (1.01-1.18)
All	9897	1.11 (1.09-1.14)	1408	1.04 (0.98-1.09)	666	1.00 (0.93-1.08)	788	1.08 (1.00-1.16)	797	1.33 (1.24-1.43)	2251	1.08 (1.05-1.11)

Rate ratios (RR) are adjusted for age, sex, education, province, and (where appropriate) smoking; reference category is regular drinkers who consume <1 bottle of rum per week.

One 35cl bottle of rum is equivalent to 110g of pure alcohol. Exclusions and conventions are as per Table 1.

⁎ Linear associations of usual alcohol consumption vs all-cause mortality among regular (ie, at least weekly) drinkers.

There was no evidence that the strength of this association varied by age (35–69 years vs 70–79 years), sex or smoking status (ever vs never), although the findings were less robust in women than in men, and in never than ever smokers (Table 2; appendix p 10). Neither was there evidence that the risk of all-cause mortality in occasional drinkers differed from that among regular drinkers in the lowest category of weekly alcohol consumption. Non-drinkers (which included never drinkers and ex-drinkers) had about 10% higher risk of all-cause mortality than regular drinkers in in the lowest weekly alcohol category (RR 1.11 [95% CI 1.09-1.14]).

In cause-specific analyses (Fig. 2, Table 3), there were positive and approximately log-linear associations of weekly rum consumption with risk of mortality from various diseases, including cancer (RR [95%CI] per 35cl bottle of rum per week 1.07 [1.03-1.12]), vascular disease (1.05 [1.00-1.10]), liver cirrhosis (1.32 [1.12-1.55]), and external causes (1.15 [1.04-1.28]). Overall, about one tenth of the mortality among weekly drinkers (255 [11%] of 2251 deaths) was attributable to alcohol consumption: 27% of this excess mortality was accounted for by cancer deaths, 21% by vascular deaths, 15% by external causes, 14% by deaths from liver cirrhosis, 7% by respiratory deaths, and 16% by other causes (appendix p 11).

**Table 3:** Alcohol consumption vs cause-specific mortality at ages 35-79 years in Cuba.

	Non-drinkers		Occasional drinkers		Regular drinkers (35cl bottles of rum per week)						RR per 35cl bottle of rum per week*	
					<1		1-<3		≥3			
	Deaths	RR(95%CI)	Deaths	RR(95%CI)	Deaths	RR(95%CI)	Deaths	RR(95%CI)	Deaths	RR(95%CI)	Deaths	RR(95%CI)
Vascular	3884	1.10 (1.06-1.15)	518	1.00 (0.92-1.09)	256	1.00 (0.88-1.13)	284	1.04 (0.92-1.17)	261	1.19 (1.05-1.34)	801	1.05 (1.00-1.10)
Ischaemic heart disease	1893	1.18 (1.12-1.26)	269	1.08 (0.96-1.22)	123	1.00 (0.84-1.19)	138	1.04 (0.88-1.23)	125	1.16 (0.97-1.39)	386	1.04 (0.97-1.11)
Stroke	779	0.78 (0.71-0.86)	117	0.81 (0.68-0.98)	66	1.00 (0.79-1.27)	67	1.01 (0.79-1.29)	59	1.13 (0.86-1.46)	192	1.03 (0.94-1.14)
Other	1212	1.28 (1.19-1.39)	132	1.02 (0.86-1.22)	67	1.00 (0.79-1.27)	79	1.08 (0.87-1.35)	77	1.32 (1.05-1.66)	223	1.08 (0.99-1.17)
Cancer	2984	1.01 (0.96-1.06)	503	1.08 (0.99-1.18)	228	1.00 (0.88-1.14)	269	1.04 (0.92-1.18)	276	1.29 (1.14-1.45)	773	1.07 (1.03-1.12)
Lung	671	0.93 (0.84-1.02)	124	0.96 (0.80-1.15)	71	1.00 (0.79-1.26)	79	0.88 (0.70-1.10)	79	1.00 (0.80-1.26)	229	1.01 (0.93-1.10)
Upper aero-digestive	171	0.60 (0.51-0.72)	36	0.61 (0.44-0.86)	35	1.00 (0.72-1.39)	44	0.95 (0.71-1.28)	79	1.93 (1.53-2.44)	158	1.23 (1.11-1.36)
Other	2142	1.17 (1.11-1.24)	343	1.28 (1.15-1.43)	122	1.00 (0.84-1.19)	146	1.14 (0.96-1.34)	118	1.15 (0.95-1.38)	386	1.03 (0.96-1.10)
Other medical	2500	1.27 (1.20-1.34)	306	1.05 (0.93-1.17)	140	1.00 (0.85-1.18)	173	1.15 (0.99-1.33)	192	1.56 (1.35-1.81)	505	1.13 (1.07-1.19)
Respiratory	985	1.26 (1.16-1.37)	110	1.09 (0.90-1.32)	55	1.00 (0.77-1.30)	61	0.98 (0.76-1.26)	71	1.38 (1.09-1.75)	187	1.10 (1.01-1.21)
Liver cirrhosis	106	0.92 (0.71-1.18)	16	0.79 (0.48-1.29)	10	1.00 (0.54-1.86)	23	2.03 (1.34-3.07)	32	3.48 (2.41-5.02)	65	1.32 (1.12-1.55)
Other	1409	1.31 (1.22-1.41)	180	1.05 (0.91-1.21)	75	1.00 (0.80-1.25)	89	1.15 (0.93-1.42)	89	1.44 (1.16-1.78)	253	1.10 (1.01-1.19)
External	496	1.21 (1.08-1.36)	79	1.05 (0.84-1.32)	39	1.00 (0.73-1.37)	56	1.24 (0.95-1.62)	66	1.74 (1.36-2.24)	161	1.15 (1.04-1.28)
Ill-defined	33	1.18 (0.75-1.83)	2	0.34 (0.08-1.35)	3	1.00 (0.32-3.09)	6	1.55 (0.69-3.49)	2	0.57 (0.14-2.31)	11	0.82 (0.51-1.33)
All	9897	1.11 (1.09-1.14)	1408	1.04 (0.98-1.09)	666	1.00 (0.93-1.08)	788	1.08 (1.00-1.16)	797	1.33 (1.24-1.43)	2251	1.08 (1.05-1.11)

Rate ratios (RR) are adjusted for age, sex, education, province, and smoking; reference category is regular drinkers who consume <1 bottle of rum per week. One 35cl bottle of rum is equivalent to 110g of pure alcohol. Exclusions and conventions are as per Table 1.

⁎ Linear association of usual alcohol consumption vs all-cause mortality among regular (ie, at least weekly) drinkers.

For men aged 35-69 years, the uniformly age-standardised annual death rate per 1000 men was 10.3 (95% CI 9.4–11.2) in the highest rum consumption category, 8.4 (7.6-9.2) in the middle category and 7.8 (6.9-8.7) in the lowest category; for women of age 35–69 years, the corresponding rates in each category were 6.3 (4.6-8.9), 4.4 (3.2-6.0) and 4.1 (3.3-5.2). These uniformly age-standardised annual death rates translate into 35-year mortality risks (ie, risk of death between ages 35-69 years) for high, middle and low levels of self-reported rum consumption of 30% (95% CI 28-32), 26% (23-28), and 24% (22-26) for men, and 20% (15–27), 14% (11–19), and 13% (11–17), for women. In analyses stratified by smoking habits (as most drinkers were also smokers), the absolute risks associated with higher levels of alcohol consumption were even more extreme among smokers (Table 4).

**Table 4:** Absolute risk of all-cause mortality at ages 35–69 years in Cuba, by sex, smoking, and level of alcohol consumption.

	Deaths	Annual mortality rate per 1000 (95% CI)	35-year risk of death, % (95% CI)*
**Men, current smokers (2271 deaths)**			
Non-drinker	980	9.1 (8.6-9.8)	27.4 (26.0-28.9)
Occasional drinker	297	9.1 (8.2-10.2)	27.4 (24.8-30.1)
Regular: <1 bottle per week	211	9.0 (7.8-10.2)	26.9 (23.9-30.1)
Regular: 1-<3 bottles per week	371	10.3 (9.3-11.5)	30.3 (27.8-33.1)
Regular: ≥3 bottles per week	412	12.6 (11.5-14.0)	35.7 (33.1-38.7)
**Men, not current smokers (1400 deaths)**			
Non-drinker	921	6.5 (6.2-70)	20.5 (19.5-21.7)
Occasional drinker	181	6.0 (5.2-7.0)	18.9 (16.6-21.7)
Regular: <1 bottle per week	106	6.4 (5.3-7.7)	20.0 (16.9-23.6)
Regular: 1-<3 bottles per week	96	5.7 (4.7-7.0)	18.2 (15.0-21.7)
Regular: ≥3 bottles per week	96	7.9 (6.5-9.6)	24.1 (20.2-28.5)
**Women, current smokers (1331 deaths)**			
Non-drinker	1081	7.5 (7.1-7.9)	23.1 (22.0-24.3)
Occasional drinker	144	5.4 (7.4-19.9)	19.9 (17.1-22.9)
Regular: <1 bottle per week	46	4.3 (7.7-18.1)	18.1 (13.9-23.5)
Regular: 1-<3 bottles per week	32	4.3 (8.6-19.1)	19.1 (13.9-25.9)
Regular: ≥3 bottles per week	28	6.3 (13.2-27.4)	27.4 (19.7-37.0)
**Women, not current smokers (2084 deaths)**			
Non-drinker	1859	4.5 (4.3-14.4)	14.4 (13.9-15.1)
Occasional drinker	181	4.3 (3.7-5.0)	14.1 (12.2-16.0)
Regular: <1 bottle per week	28	3.2 (2.2-4.6)	10.6 (7.3-15.0)
Regular: 1-<3 bottles per week	10	3.1 (1.7-5.8)	10.4 (5.8-18.5)
Regular: ≥3 bottles per week	6	3.9 (1.8-8.7)	12.9 (6.0-26.4)

Annual mortality rates were estimated by multiplying rate ratios at age 35-69 years by a common factor so that their inverse-variance weighted average matched the Cuban national mortality rate at age 35-69 years in 2010 for men and women. One 35cl bottle of rum is equivalent to 110g of pure alcohol. Exclusions and conventions are as per Table 1.

⁎ 35-year risk of death between ages 35 and 69 years.

In sensitivity analyses (appendix pp 9,12-14), further adjustment for other potential confounders, including diabetes, occupation, marital status and body-mass index, did not materially change the associations. Neither was there any effect of further excluding those on blood pressure lowering medication or by excluding the first few years of follow-up (to further limit reverse causality), with the exception of the increased risk among non-drinkers which became progressively attenuated: in the first 5 years of follow-up, the RR for all-cause mortality among non-drinkers was 1.17 (95% CI 1.11-1.23), which attenuated to 1.04 (0.98-1.11) after excluding the first 15 years of follow-up.

## 4 Discussion

In this large prospective study of Cuban adults, higher alcohol consumption was approximately log-linearly associated with all-cause mortality throughout the range examined. Overall, each additional 35cl bottle of rum (or equivalent alcohol consumption) per week was associated with about 10% higher risk of all-cause mortality. There was no evidence of excess mortality among occasional drinkers, but mortality among non-drinkers was somewhat higher than among regular drinkers in the lowest category of weekly alcohol consumption; however, the attenuation of this association on exclusion of early follow-up indicates that reverse causality is likely to account for much of the apparent excess risk.

To our knowledge, this is the largest prospective study to assess the effect of alcohol consumption on mortality in Latin America. The study strongly reinforces evidence from elsewhere on the substantial risk of all-cause mortality with high alcohol consumption [1,2,14]. Consistent with the findings from other studies, there were strong positive associations between weekly alcohol consumption and risk of death from cancer, liver disease and external causes, but unlike many studies there was no protective effect on vascular mortality [2].

Prospective studies have tended to find higher mortality among both never drinkers and ex-drinkers, relative to those that drink low levels of alcohol [[15], [16], [17]]. As this study was not able to differentiate never drinkers from ex-drinkers, and it is unclear as to whether the excess risk among non-drinkers was the result of higher mortality among both of these groups. The substantial attenuation in the strength of the association among non-drinkers on exclusion of the first years of follow-up would indicate that reverse causality (whereby ill-health determined drinking status among ex-drinkers, never drinkers, or both) is likely to account for much of the apparent excess risk in this group. The causality of the excess risk among never drinkers and ex-drinkers in other populations has been further challenged by recent findings from studies using genetic epidemiology to limit the effects of bias from confounding and reverse causality [15,18,19].

The modest excess risk of vascular mortality at high levels of alcohol consumption is consistent with that generally described in high-income countries [1]. Unfortunately, there were insufficient events to reliably assess the strength of association with specific causes of vascular death. A recent large meta-analysis of prospective studies described contrasting associations of alcohol consumption with vascular disease subtypes: there were positive associations with stroke, coronary disease excluding myocardial infarction, heart failure, and aortic aneurysm, but an inverse association with myocardial infarction [1]. Differences in the associations between alcohol consumption and all vascular mortality in different populations may well reflect variation in the proportions of vascular deaths from these subtypes.

Alcohol consumption was strongly related to cancer mortality in the present study. Other large-scale prospective studies have described positive associations of alcohol consumption with cancer of the larynx, oral cavity and pharynx, oesophagus, liver, breast, and rectum, and inverse associations with cancer of the thyroid, non-Hodgkin's lymphoma, and renal carcinoma [3]. Although the present study lacked sufficient events to assess the strength of the association with some of these cancers, there were strong positive associations with upper aero-digestive cancers (which accounted for about half of the excess cancer deaths among weekly drinkers), and the lack of association with lung cancer indicates, reassuringly, that there was little evidence of residual confounding from smoking.

Overall, after taking appropriate precautions to avoid bias due to confounding and reverse causality (whereby life-threatening disease can itself change drinking habits), the present study found all-cause mortality was linearly related to regular weekly alcohol consumption, with no strong evidence of excess risks among occasional or non-drinkers, or of a protective effect from moderate drinking, relative to the lowest category of weekly rum consumption (<1 bottle per week, equivalent to ∼14 UK units of alcohol per week). These findings are consistent with a recent meta-analysis in high-income countries which reports a linear relationship between all-cause mortality and weekly alcohol intake, with a threshold for the lowest risk at about 100g of alcohol per week (equivalent to 12.5 UK units of alcohol per week, or just less than one 35cl bottle of rum per week) [1]. A meta-analyses of the global literature described similar findings with the risks of all-cause mortality increasing linearly with daily alcohol consumption, but with no evidence of a threshold below which alcohol was not associated with mortality [2].

This study has several keys strengths. It is the largest study to explore the associations between alcohol consumption and risk of premature death in Latin America; previous studies have had substantially fewer participants [[20], [21], [22]]. The study conducted a large resurvey to allow reliable estimates of the associations with long-term average alcohol consumption among regular drinkers. The resurvey also identified the poor reproducibility of occasional drinkers at baseline, indicating that the risks among occasional drinkers in this cohort cannot be extrapolated reliably to long-term occasional (ie, less than weekly) drinkers; a finding that may also be relevant to other cohorts. Furthermore, baseline occasional drinkers had very different patterns of drinking at resurvey to regular drinkers in the lowest category of alcohol consumption, highlighting the importance of not combining these groups in this cohort. In addition, the baseline survey collected information on prior disease to limit the effect of reverse causality, and on a range of factors to allow adjustment for major potential confounders.

It is a limitation of this study that alcohol was self-reported; previous studies have found heavy drinkers, in particular, tend to under-report their drinking. Participants were also asked to report rum and beer consumption only, and although consumption of other types of alcohol was rare at the time of the baseline survey, such information would have given a more complete assessment of alcohol consumption. Furthermore, as participants reported weekly rum consumption and weekly beer consumption separately, it was not possible to assess the frequency (in total number of days per week) that alcohol was consumed; or the total amount consumed on a given occasion to assess the effect of heavy episodic (binge) drinking. Neither was it possible to assess the effect of weekly drinking at very low levels of alcohol consumption, or to extrapolate the findings reliably to those outside the age range examined, such as those over age 80 years. Finally, as almost all drinkers drank rum, it was not possible to assess reliably the effects of beer drinking independently of rum.

National guidelines on alcohol consumption vary markedly between countries [23]. The current UK guidelines advise both men and women to limit weekly alcohol consumption to 14 units of alcohol (equivalent to 112g per week; one UK unit equals 10ml or 8 grams of pure alcohol) and to spread intake over three or more days a week [24]. Cuba does not have official guidelines on alcohol consumption, but policy documents tend to refer to US alcohol consumption guidelines, which advise no more than one standard US drink per day for women (equivalent to 98g per week) and two standard drinks per day for men (196g per week) [25].

The total alcohol consumption per capita in Cuba is lower than average for the WHO Region of the Americas [23]. In 2016, the estimated annual consumption per capita (adults over age 15 years) was 6.1 litres of pure alcohol (10.2 litres in men and 1.9 litres in women, respectively), as compared to 8.0 litres for the WHO Region of the Americas. Also, the prevalence of alcohol use disorders in Cuba is slightly lower than the WHO regional average: 6.6% of adults over age 15 years in Cuba, compared with 8.2% in the WHO Region of the Americas [23].

Addressing the 2015 UN Sustainable Development Goal of the prevention and treatment of harmful alcohol use, and the more specific target to reduce by one third premature mortality from non-communicable diseases by 2030, will require a strengthening of alcohol control policies in Cuba. Cost-effective interventions shown to reduce harmful use of alcohol consumption include restrictions on alcohol advertising, restrictions on physical availability of retailed alcohol, and increased taxes on alcohol sales [26,27]. Furthermore, efforts should be made to routinely monitor alcohol-related mortality [28].

This prospective study provides reliable evidence on the relation between alcohol consumption and cause-specific mortality in Cuba. Together with evidence from elsewhere, the findings support limits to alcohol consumption that are lower than present recommendations in Cuba, and reinforce the importance of alcohol to non-communicable disease prevention in the region.

## Author Contributions

NAR, RP and SL directed the study. NAR was responsible for field supervision. NAR, PV-P, EL-V, MCM, SBC, JMMR, OJHL, MAMM, IAA, FAE, MDG, NRM and MCA collected data. NAR, DMS, SR, JAB, PS, BL and SL were responsible for data analysis, interpretation, and reporting. NAR, DMS, SR, BL and SL drafted the article, which was revised by all authors. NAR, BL, DMS, and SR contributed equally as first authors.

## Data sharing statement

Data access requests are welcomed. Details of how to access the Cuba Prospective Study data can be found at www.ctsu.ox.ac.uk/research/the-cuba-prospective-study or by contacting cuba.prospective.study@ndph.ox.ac.uk.

## Funding

This study was funded by core support from the UK Medical Research Council (MRC), British Heart Foundation (BHF), and Cancer Research UK (CRUK) to the Clinical Trial Service Unit and the MRC Population Health Research Unit, both now in the Nuffield Department of Population Health (NDPH), University of Oxford (Oxford, UK). Support was also received from the US Centers for Disease Control and Prevention (CDC) Foundation (with support from Amgen). BL acknowledges support from UK Biobank, the National Institute for Health Research Biomedical Research Centre (Oxford, UK), and the BHF Centre of Research Excellence (Oxford, UK).

## Declaration of Competing Interest

SL reports grants from the Medical Research Council (MRC) during the conduct of the study, and research funding from the US Centers for Disease Control and Prevention Foundation (with support from Amgen). JE reports research funding to the University of Oxford from Boehringer Ingelheim outside the submitted work. All other authors declare no competing interests.

## Appendix Supplementary materials

Image, application 1
