# Supplementary material for: Alcohol consumption and cause-specific mortality in Cuba: prospective study of 120 623 adults
Source: eClinicalMedicine. 2021 Mar 17;33:100692. doi: 10.1016/j.eclinm.2020.100692 (PMC7980059; doi:10.1016/j.eclinm.2020.100692)

Alcohol consumption and cause-specific mortality in Cuba: prospective study of 120 623 adults

Supplementary Appendix, Table of Contents

	Page
**Questionnaire** (Spanish)	2
**Questionnaire** (English translation)	3
**Table S1.** Mortality endpoints and ICD codes	4
**Table S2.** Number of participants excluded from mortality analyses, by sex	5
**Table S3.** Frequency of alcohol consumption among regular drinkers included in the main analyses, by sex and type of alcohol consumed	6
**Table S4.** Alcohol consumption at baseline and resurvey (mean 8.6 years after recruitment), among 20 593 participants	7
**Table S5.** Alcohol consumption versus all-cause mortality at ages 35-79 years in Cuba, among regular (ie, at least weekly) drinkers, with progressive adjustment for potential confounders	8
**Figure S1.** Alcohol consumption versus all-cause mortality at ages 35-79 years in Cuba, during follow-up years <5, 5-<10, 10-<15, and 15 onwards	9
**Figure S2.** Alcohol consumption versus all-cause mortality in Cuba, by age, by sex, and by smoking	10
**Table S6.** Observed and expected numbers of deaths from specific causes at ages 35-79 years in Cuba, by weekly alcohol consumption	11
**Figure S3.** Alcohol consumption versus all-cause, vascular, cancer and other mortality at ages 35-79 years in Cuba, with additional adjustment for other potential confounders	12
**Figure S4.** Alcohol consumption versus all-cause, vascular, cancer and other mortality at ages 35-79 years in Cuba, excluding first two years of follow-up	13
**Figure S5.** Alcohol consumption versus all-cause, vascular, cancer and other mortality at ages 35-79 years in Cuba, excluding particpants taking anti-hypertensives	14

ESTUDIO CUBANO SOBRE TABACO Y SALUD

Provincia No. ☐ 1-9 Municipio No. ☐☐ 0-99 Area de Salud No. ☐ 0-9

№ 17524 A

Carnet de Identidad No. |_|_|_|_|_|_|—|_|_|_|_|_|
Fecha de nacimiento (a.m.d) + 5 números
(11 números: excluir si no tiene C.I. de 6 + 5 números)

Nombre de la persona y dirección: ____________________
Calle No. Teléfono

Nombre del familiar y dirección: ____________________
Calle No. Municipio Provincia Teléfono

LETRA DE MOLDE, POR FAVOR

Sexo (1 = masc, 2 = fem.) ☐ 1 - 2

Color de piel (1 = blanco, 2 = negro, 3 = mestizo, 4 = otra) ☐ 1 - 4

Estado social (1 = soltero/viudo, 2 = casado) ☐ 1 - 2

Educación completada (0 = ninguno*, 1 = primaria, 2 = secundaria, 3 = técnico medio, 4 = obrero calificado, 5 = pre-universitario, 6 = universitario) ☐ 0 - 6

Categoría ocupacional (0 = ninguno*, 1 = dirigente, 2 = profesional, 3 = técnico, 4 = de servicio, 5 = agrícola, 6 = industria, 7 = militar) ☐ 0 - 7

* Si es cero (Ej. ama de casa, jubilado o desempleado), diga si ha trabajado alguna vez. ☐ 0 - 7

Quisiera medir su presión arterial mientras esta sentado, hacerle unas pocas preguntas sobre tabaco y alcohol, y medir su PA de nuevo.

Presión arterial (una sola toma, sentado): |_|_|_|_| / |_|_|_|_| PAS / PAD

Ahora, TABACO: ¿Ha fumado alguna vez regularmente (Ej. en la mayoría de los días)?
SI NO, vaya directamente a las preguntas sobre ALCOHOL. ☐ S=Sí, N=No

¿A QUE EDAD APROXIMADAMENTE COMENZO USTED A FUMAR REGULARMENTE? ☐☐ años de edad

¿Desde entonces ha dejado de fumar alguna vez por un año o más? ☐ S=Sí, N=No

¿Aproximadamente cuántos cigarrillos por día se fumó usted el último mes? (0 = ninguno) ☐☐ diariamente

¿Aproximadamente cuántos tabacos por día se fumó usted el último mes? (0 = ninguno) ☐☐ diariamente

¿Si no fumó el último mes, a que edad dejó usted de fumar? (en blanco si no es aplicable) ☐☐ años de edad

Ahora me gustaría hacerle algunas preguntas sobre ALCOHOL:

¿Aproximadamente cuántos días a la semana toma usted ron u otra bebida fuerte? (0 = cero días o usualmente menos de una vez por semana) ☐ 0 - 7

¿El día que usted toma ron (u otra bebida fuerte) cuántas líneas se toma? ☐☐ líneas

¿Aproximadamente cuántos días a la semana toma usted cerveza? (0 = cero días o usualmente menos de una vez por semana) ☐ 0 - 7

¿El día que usted toma cerveza, cuántas botellas se toma (aprox.) ☐☐ botellas

Finalmente, algunos aspectos médicos: Quisiera preguntarle sobre algunas enfermedades. Lo que quiero conocer es si usted alguna vez ha sido tratado por alguna de estas enfermedades. Si no lo ha sido, conteste No (N).

Asma	☐ S/N	A.V.E. (definitivo/probable)	☐ S/N	Cirrosis	☐ S/N
Enfisema (E.P.O.C.)	☐ S/N	Infarto miocardio "	☐ S/N	Enf. renal crónica	☐ S/N
Diabetes	☐ S/N	Angina pectoris "	☐ S/N	Cáncer de piel	☐ S/N
Ulcera péptica	☐ S/N	Hipertensión arterial	☐ S/N	Otro cáncer*	☐ S/N

¿*Si Otro Cáncer = Sí, dónde le comenzó el cáncer? ☐ 0-9

(1 = boca o faringe, 2 = laringe, 3 = pulmón, 4 = esófago, 5 = estómago, 6 = páncreas, 7 = cuello útero, 8 = vejiga, 9 = mama, 0 = otro, más de un sitio, o no conocido)

¿Esta usted tomando alguna medicina regularmente desde hace más de un mes? Si es Sí: ¿Qué medicinas esta tomando? (Marque con X)

Digoxina ☐	Nitratos ☐	Hipoglic. oral ☐	Anticoncept. ☐	Ca. Antag. ☐
Beta B. ☐	Inh. ACE ☐	Insulina ☐	Antidepres. ☐	Broncodilat. ☐
Diurético ☐	ASA ☐	Esteroides ☐	Ansiolítico ☐	Antiácido/H2 ☐

MUCHAS GRACIAS POR SUS RESPUESTAS: Ahora necesito tomarle de nuevo su presión arterial. Repita la toma de PA (una sola toma sentado): |_|_|_|_| / |_|_|_|_| PAS/PAD

¿Cuál es la última cifra PA que tiene en su historia clínica antes de hoy?

Fecha de la última PA (a.m.d) |_|_|_|_|_|_| & |_|_|_|_| / |_|_|_|_| PAS/PAD

¿Cuál es su *TALLA* aproximada? (medirlo o preguntarlo) |_|_|_|_| cm

¿Cuál es su *PESO* aproximado? (pesarlo o preguntarlo) |_|_|_|_| kg

Fecha de la entrevista |_|_|_|_|_|_| (a.m.d)

Por favor chequee TODA la encuesta ¡MUCHAS GRACIAS!

Calidad de la cooperación (1 = pobre, 2 = regular, 3 = buena) ☐ 1 - 3

Nombre y apellidos del entrevistador: ____________________

¿Algún comentario? ....

PARA LA SIGUIENTE PERSONA, UTILICE EL REVERSO DE ESTA PAGINA →

CUBAN STUDY OF TOBACCO AND HEALTH: PROSPECTIVE

Province Nº [ ] 1-9 Municipality Nº [ ][ ] 0-99 Health Area Nº [ ] 0-9 Identity Card Nº [ ][ ][ ][ ][ ][ ]-[ ][ ][ ][ ][ ] Date of birth (y,m,d)

Name of interviewee:

Address: Street Nº Municipality Province Telephone

Name of friend:

Address: Street Nº Municipality Province Telephone

CAPITAL LETTERS, PLEASE!

Sex (1=masc, 2=fem) [ ] 1-2

Skin colour (1=white, 2=black, 3=mixed, 4=other) [ ] 1-4

Marital status (1=single/widowed, 2=married) [ ] 1-2

Education completed (0=none, 1=primary, 2=secondary, 3=technician, 4=skilled worker, 5=high school, 6=university) [ ] 0-6

Occupational category (0=none, 1=manager, 2=professional, 3=technician, 4=service worker, 5=agricultural, 6=industrial, 7=military; if housewife, retired or unemployed, we would like to know if you were employed in the past) [ ] 0-7

([ ] 0-7)

What I'd like to do is measure your blood pressure while you are sitting down, ask you a few questions about tobacco and alcohol, and then do your blood pressure again. Blood pressure (single measurement, seated): [ ][ ][ ] / [ ][ ][ ] SBP / DBP

Now, TOBACCO: Have you EVER smoked regularly (i.e. on most days for at least a year)? If NO, then go directly to the ALCOHOL questions [ ] **Y=Yes, N=No**

AT ABOUT WHAT AGE DID YOU FIRST START SMOKING TOBACCO REGULARLY? [ ][ ] years of age

Since then, have you ever STOPPED smoking for a year or more? [ ] Y=Yes, N=No

About how many cigarettes a day did you smoke last month? (0=none) [ ][ ] daily

About how many cigars a day did you smoke last month? (0=none) [ ][ ] daily

If NO SMOKING last month, at about what age did you last stop smoking? (blank = not applicable) [ ][ ] years of age

Now I'd like to ask you some questions about ALCOHOL:

About how many days a week would you drink any rum or other spirits? (0=none or usually less than once a week) [ ] 0-7

On a day when you drink rum (or other spirits), about how many glasses ("lineas") would you drink? [ ][ ] lineas

About how many days a week would you drink beer? (0=none or usually less than once a week) [ ] 0-7

On a day when you do drink beer, about how many bottles would you drink? [ ][ ] bottles

Finally, MEDICAL MATTERS: I'd like to ask about various different illnesses. What I want to know for each illness is whether you've ever had medical attention for it. If you've never heard of the illness, then just say NO.

Asthma	[ ] Y/N	Stroke (definite/probable)	[ ] Y/N	Cirrhosis of the liver	[ ] Y/N
Emphysema/C.O.P.D.	[ ] Y/N	Myocardial infarction "	[ ] Y/N	Chronic kidney disease	[ ] Y/N
Diabetes	[ ] Y/N	Angina pectoris "	[ ] Y/N	Skin cancer	[ ] Y/N
Peptic ulcer	[ ] Y/N	Hypertension	[ ] Y/N	Other cancer*	[ ] Y/N

*If Other cancer, where in your body did the cancer start? [ ] 0-9
(1=mouth, 2=larynx, 3=lung, 4=oesphagus, 5=stomach, 6=pancreas, 7=cervix, 8=bladder, 9=breast, 0=other, more than 1 site, or not known)

Have you been taking any regular medication for more than 1 month? If Yes: What such medicines are you now taking? (Mark with an **X**)

Digoxin	[ ]	Nitrates	[ ]	Oral hypoglycaemic	[ ]	O.Contraceptive	[ ]	Ca. antagonist	[ ]
Beta blocker	[ ]	ACE inhibitor	[ ]	Insulin	[ ]	Antidepressant	[ ]	Bronchodilator	[ ]
Diuretic	[ ]	Aspirin	[ ]	Steroids	[ ]	Tranquillisers	[ ]	Antacid/H2	[ ]

THANK YOU VERY MUCH FOR YOUR ANSWERS. Now we just need one more blood pressure. Repeat blood pressure (single measurement, seated) [ ][ ][ ] / [ ][ ][ ] PAS / PAD

What was the last blood pressure in the patient's medical record **before** today?

Date of last BP (y,m,d) [ ][ ][ ][ ][ ][ ] & [ ][ ][ ] / [ ][ ][ ] PAS / PAD

What is your approximate **HEIGHT** (either ask or measure)? [ ][ ][ ] cm

What is your approximate **WEIGHT** (either ask or measure)? [ ][ ][ ] kg

Date of interview [ ][ ][ ][ ][ ][ ] (y,m,d)

Quality of co-operation (1=poor, 2=fair, 3=good) [ ] 1-3

Please check that **ALL** the questions have been answered, so that there is no need to write back to you for extra details. **MANY THANKS!**

Name of interviewer: please PRINT

Any comments: ....

Table S1: Mortality endpoints and ICD codes

Endpoint name	ICD-9 codes	ICD-10 codes
**Vascular**	390-459, 798	I00-I99, R96
Ischaemic heart disease	410-414	I20-I25
Stroke	430-431, 433-434, 436	I60-I61, I63-I64
Other	390-409, 415-429, 432, 435, 437-459, 798	I00-I19, I26-I59, I62, I65-I99, R96
**Neoplastic**	140-208	C00-C97
Lung cancer	162	C33-C34
Upper aero-digestive cancer	140-150, 161	C00-C15, C32
Other	151-160, 163-208	C16-C31, C35-C97
**Other medical**	000-1002 excl (390-459, 798, 140-208, 800-1002, 780-797, 799)	A00-Y89 excl (I00-I99, R96, C00-C97, S00-Y89, R00-R95, R98-R99)
Respiratory	460-519, 010-012	J00-J98, A15-A16
Liver cirrhosis	571	K70, K74
Other	000-1002 excl (390-459, 798, 140-208, 460-519, 010-012, 571, 800-1002, 780-797, 799)	A00-Y89 excl (I00-I99, R96, C00-C97, J00-J98, A15-A16, K70, H74, S00-Y89, R00-R95, R98-R99)
**External**	800-1002	S00-Y89
**Ill-defined**	780-797, 799	R00-R95, R98-R99
**All causes**	000-1002	A00-Y89

Table S2: Number of participants excluded from mortality analyses, by sex

	Men	Women	All
**Number of participants recruited**	64 743 (100%)	81 813 (100%)	146 556 (100%)
**Reasons for exclusion (not mutually exclusive):**			
**Prior disease***	9061 (14.0%)	10 850 (13.3%)	19 911 (13.6%)
**Alcohol variables**			
Missing values	152 (0.2%)	20 (0.02%)	172 (0.1%)
Incomplete information	211 (0.3%)	92 (0.1%)	303 (0.2%)
**Missing values in covariates**			
Age	0 (0%)	0 (0%)	0 (0.0%)
Sex	0 (0%)	0 (0%)	0 (0.0%)
Province	0 (0%)	0 (0%)	0 (0.0%)
Level of education	61 (0.1%)	103 (0.1%)	164 (0.1%)
Smoking	0 (0%)	0 (0%)	0 (0.0%)
**No follow-up at ages 35-79 years**			
Age 80 years or over at baseline	3333 (5.1%)	4156 (5.1%)	7489 (5.1%)
**Total participants excluded**	**11 814 (18.2%)**	**14 119 (17.3%)**	**25 933 (17.7%)**
**Total participants in main analyses**	**52 929 (81.8%)**	**67 694 (82.7%)**	**120 623 (82.3%)**

*Prior history at baseline of myocardial infarction, angina, stroke, chronic obstructive pulmonary disease, cirrhosis of the liver, chronic kidney disease, any cancer (including skin), or peptic ulcer.

Table S3. Frequency of alcohol consumption in regular drinkers included in the main analyses, by sex and type of alcohol consumed

	Days per week of alcohol consumption							
	1	2	3	4	5	6	7	All
**Men**								
Rum only drinkers	2520 (30%)	2193 (26%)	1366 (16%)	557 (7%)	428 (5%)	170 (2%)	1282 (15%)	8516 (100%)
Beer only drinkers	486 (56%)	219 (25%)	76 (9%)	21 (2%)	16 (2%)	8 (1%)	38 (4%)	864 (100%)
Drinkers of both rum and beer								
Rum	2494 (41%)	1643 (27%)	800 (13%)	317 (5%)	239 (4%)	93 (2%)	455 (8%)	6041 (100%)
Beer	3313 (55%)	1556 (26%)	556 (9%)	185 (3%)	152 (3%)	45 (1%)	234 (4%)	6041 (100%)
All (highest frequency of rum or beer)	2185 (36%)	1695 (28%)	906 (15%)	365 (6%)	283 (5%)	109 (2%)	498 (8%)	6041 (100%)
**Women**								
Rum only drinkers	547 (49%)	245 (22%)	139 (12%)	48 (4%)	33 (3%)	17 (2%)	87 (8%)	1116 (100%)
Beer only drinkers	385 (73%)	85 (16%)	30 (6%)	4 (1%)	4 (1%)	4 (1%)	16 (3%)	528 (100%)
Drinkers of both rum and beer								
Rum	851 (61%)	291 (21%)	119 (8%)	44 (3%)	21 (1%)	16 (1%)	59 (4%)	1401 (100%)
Beer	951 (68%)	273 (19%)	79 (6%)	37 (3%)	12 (1%)	10 (1%)	39 (3%)	1401 (100%)
All (highest frequency of rum or beer)	792 (57%)	301 (21%)	140 (10%)	65 (5%)	20 (1%)	18 (1%)	65 (5%)	1401 (100%)

Exclusions and conventions are as per table 1.

Table S4. Alcohol consumption at baseline and resurvey (mean 8.6 years after recruitment), among 20 593 participants*

	Non-drinkers	Occasional drinkers	Regular drinkers (35cl bottles of rum per week)		
			<1	1-<3	≥3
**Participants, at baseline**	18 298	148	451	804	892
**Alcohol consumption at resurvey, N (%)**					
Non-drinkers	17 936 (98)	69 (47)	138 (31)	216 (27)	212 (24)
Occasional drinkers	2 (0.01)	3 (2)	1 (0.2)	0 (0)	0 (0)
Regular drinkers, 35cl bottles of rum per week					
<1	118 (0.6)	46 (31)	302 (67)	30 (4)	37 (4)
1-<3	141 (0.8)	14 (9)	6 (1)	537 (67)	75 (8)
≥3	101 (0.6)	16 (11)	4 (0.9)	21 (3)	568 (64)
**Mean alcohol consumption (35cl bottles/week), mean (SD)**					
At baseline, all participants	0 (0)	0 (0)	0.6 (0.3)	1.9 (0.5)	6.4 (3.3)
At resurvey, all participants	0.05 (0.5)	0.9 (1.7)	0.5 (0.8)	1.5 (1.8)	4.2 (4.2)
At baseline, regular drinkers at both baseline and resurvey	-	-	0.6 (0.3)	1.9 (0.5)	6.5 (3.4)
At resurvey, regular drinkers at both baseline and resurvey	-	-	0.7 (0.8)	2.1 (1.7)	5.5 (4.0)

*Exclusions and conventions are as per table 1, with further exclusion of participants with missing or incomplete information on alcohol consumption at resurvey

Table S5. Alcohol consumption versus all-cause mortality at ages 35-79 years in Cuba, among regular (ie, at least weekly) drinkers, with progressive adjustment for potential confounders

		RR per 35cl bottle of rum per week, adjusting for age, sex, and province		RR per 35cl bottle of rum per week, adjusting for age, sex, province and education		RR per 35cl bottle of rum per week, adjusting for age, sex, province, education and smoking	
	**Deaths**	**RR (95%CI)**	**Wald $\chi 2$**	**RR (95%CI)**	**Wald $\chi 2$**	**RR (95%CI)**	**Wald $\chi 2$**
**Vascular**	801	1.07 (1.03-1.12)	9.7	1.06 (1.02-1.11)	7.3	1.05 (1.00-1.10)	4.1
Ischaemic heart disease	386	1.07 (1.00-1.14)	3.9	1.06 (0.99-1.13)	3.0	1.04 (0.97-1.11)	1.4
Stroke	192	1.05 (0.96-1.16)	1.3	1.04 (0.95-1.15)	0.8	1.03 (0.94-1.14)	0.5
Other	223	1.11 (1.02-1.21)	5.5	1.10 (1.01-1.19)	4.4	1.08 (0.99-1.17)	2.9
**Cancer**	773	1.11 (1.06-1.16)	20.4	1.10 (1.06-1.16)	18.2	1.07 (1.03-1.12)	9.1
Lung	229	1.08 (0.99-1.17)	3.3	1.07 (0.98-1.16)	2.3	1.01 (0.93-1.10)	0.1
Upper aero-digestive	158	1.30 (1.18-1.43)	27.0	1.28 (1.16-1.41)	24.4	1.23 (1.11-1.36)	16.3
Other	386	1.05 (0.98-1.12)	1.9	1.05 (0.98-1.12)	1.7	1.03 (0.96-1.10)	0.8
**Other medical**	505	1.16 (1.09-1.22)	26.1	1.14 (1.08-1.21)	22.3	1.13 (1.07-1.19)	17.2
Respiratory	187	1.15 (1.05-1.26)	8.7	1.13 (1.03-1.24)	6.9	1.10 (1.01-1.21)	4.3
Liver cirrhosis	65	1.34 (1.14-1.58)	12.9	1.33 (1.13-1.56)	11.7	1.32 (1.12-1.55)	10.9
Other	253	1.12 (1.03-1.21)	7.4	1.11 (1.02-1.20)	6.3	1.10 (1.01-1.19)	5.3
**External**	161	1.18 (1.07-1.30)	10.6	1.16 (1.05-1.29)	9.0	1.15 (1.04-1.28)	7.8
**Ill-defined**	11	0.88 (0.55-1.42)	0.3	0.87 (0.54-1.40)	0.4	0.82 (0.51-1.33)	0.6
**All**	2251	1.11 (1.08-1.14)	61.3	1.10 (1.07-1.13)	51.4	1.08 (1.05-1.11)	32.3

One 35cl bottle of rum is equivalent to 110g of pure alcohol. Exclusions and conventions are as per table 1.

Figure S1. Alcohol consumption versus all-cause mortality at ages 35-79 years in Cuba, during follow-up years <5, 5-<10, 10-<15, and 15 onwards

Rate ratio (RR) adjusted for age, sex, education, province, and smoking. Exclusions and conventions as in table 1.

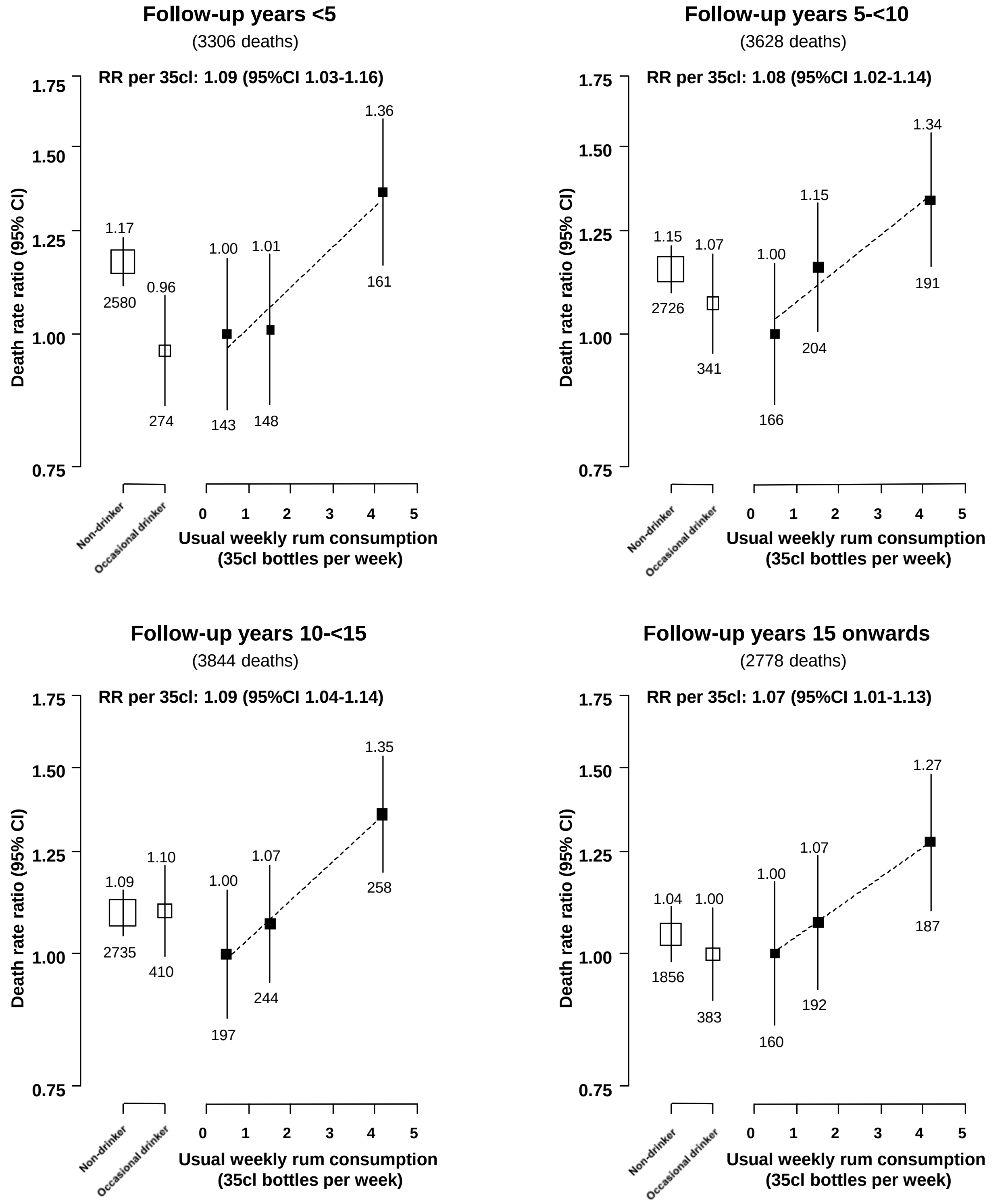

Figure S2. Alcohol consumption versus all-cause mortality in Cuba, by age, by sex and by smoking

Rate ratios (RR) adjusted for age, sex, education, province, and smoking. Exclusions and conventions as per table 1.

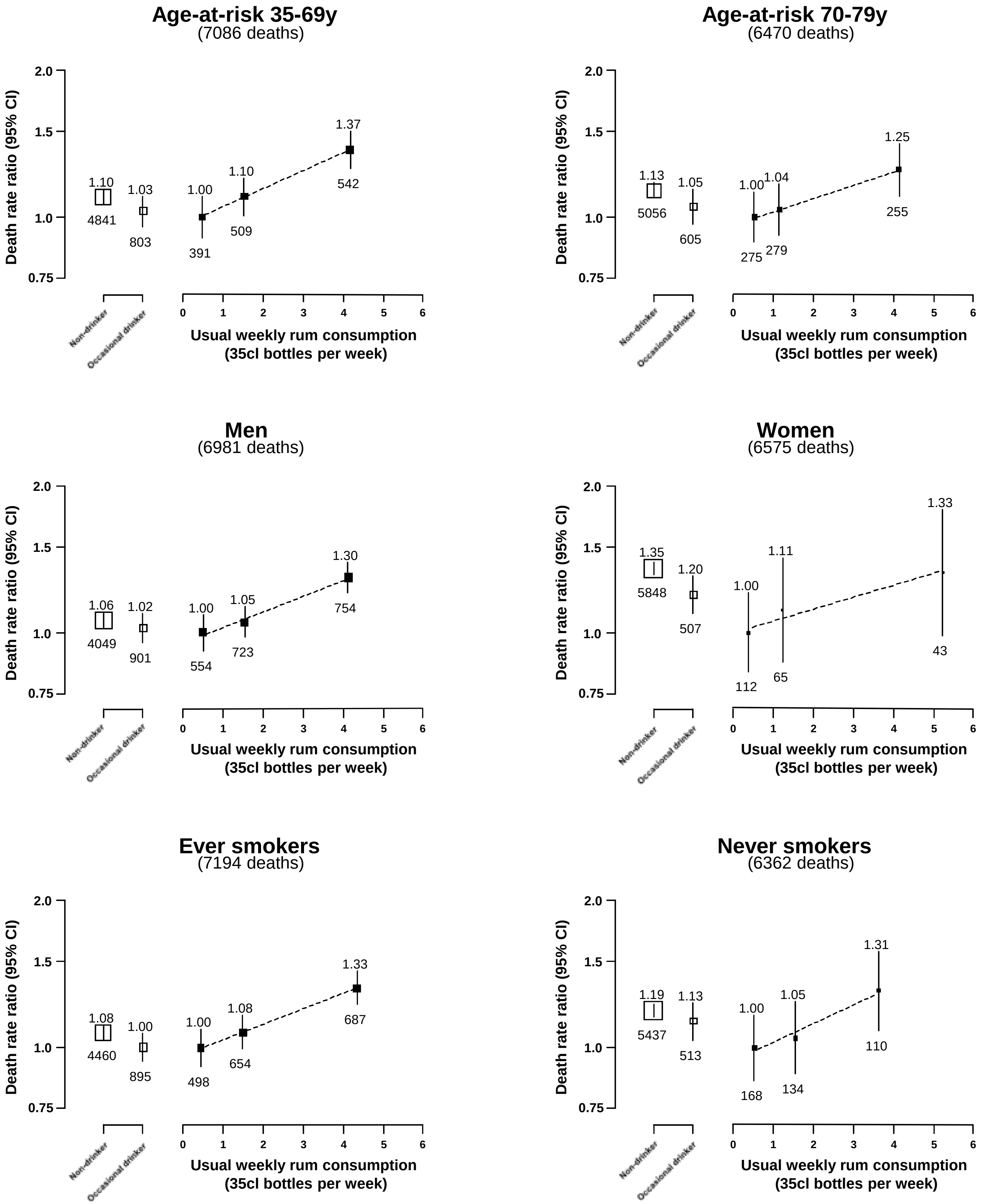

Table S6. Observed and expected numbers of deaths from specific causes at ages 35-79 years in Cuba, by weekly alcohol consumption

	At least weekly alcohol drinker (35cl bottles of rum per week)						
	1-<3 bottles		≥3 bottles		Total (≥1 bottle)		
	Observed deaths	Expected deaths	Observed deaths	Expected deaths	Observed deaths	Expected deaths	Excess Deaths
**Vascular**	284	272.2	261	218.3	545	490.5	54.5 (21%)
Ischaemic heart disease	138	132.7	125	107.8	263	240.5	22.5 (9%)
Stroke	67	66.3	59	52.2	126	118.5	7.5 (3%)
Other	79	73.1	77	58.3	156	131.5	24.5 (10%)
**Cancer***	190	174.4	197	143.5	387	317.9	69.1 (27%)
Upper aero-digestive	44	46.3	79	40.9	123	87.2	35.8 (14%)
Other	146	128.1	118	102.6	264	230.7	33.3 (13%)
**Other Medical**	173	151.0	192	122.5	365	273.4	91.6 (36%)
Respiratory	61	62.2	71	51.4	132	113.7	18.3 (7%)
Liver cirrhosis	23	11.3	32	9.2	55	20.5	34.5 (14%)
Other	89	77.4	89	61.8	178	139.2	38.8 (15%)
**External**	56	45.2	66	37.9	122	83.1	38.9 (15%)
**Ill-defined**	6	3.9	2	3.5	8	7.4	0.6 (0%)
**All**	709	646.6	718	525.7	1427	1172.3	254.7 (100%)

Expected deaths are calculated by applying the rates at ages 35-79 years (table 3) among those who reported drinking <1 bottle rum per week. Excess deaths are calculated as observed minus expected deaths. Death from vascular, cancer and other medical are sum of subcategories. Exclusions and conventions are as per table 1. *Excludes lung cancer for which there was no evidence of an association among at least weekly drinkers.

Figure S3. Alcohol consumption versus all-cause, vascular, cancer and other mortality at ages 35-79 years in Cuba, with additional adjustment for other potential confounders

Rate ratios (RR) adjusted for age, sex, education, province, smoking, occupation, marital status, diabetes, and BMI. Exclusions and conventions as per table 1.

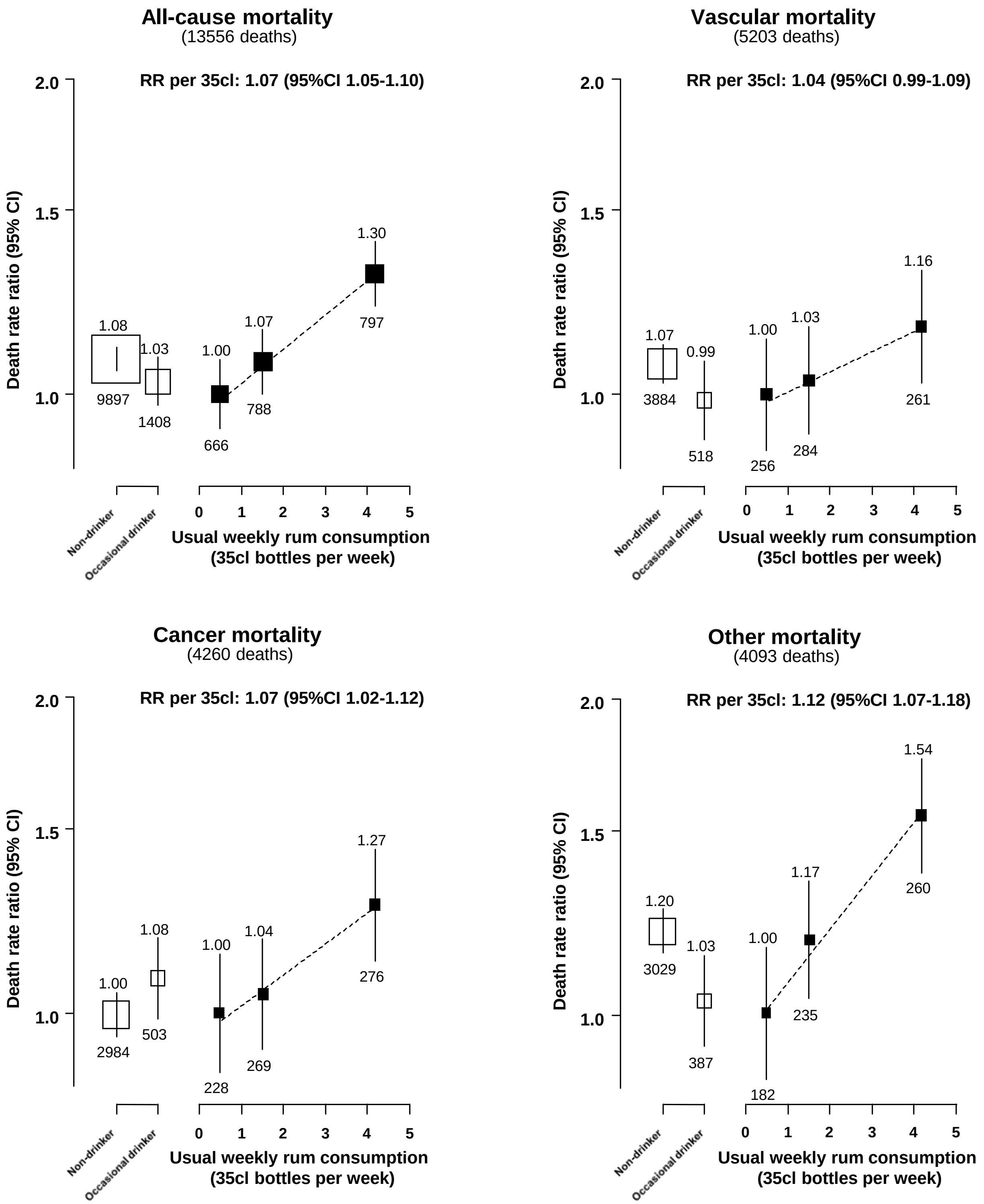

Figure S4. Alcohol consumption versus all-cause, vascular, cancer and other mortality at ages 35-79 years in Cuba, excluding first two years of follow-up

Rate ratios (RR) adjusted for age, sex, education, province, and smoking. Exclusions and conventions as per table 1, with further exclusion of the first 2 years of follow-up.

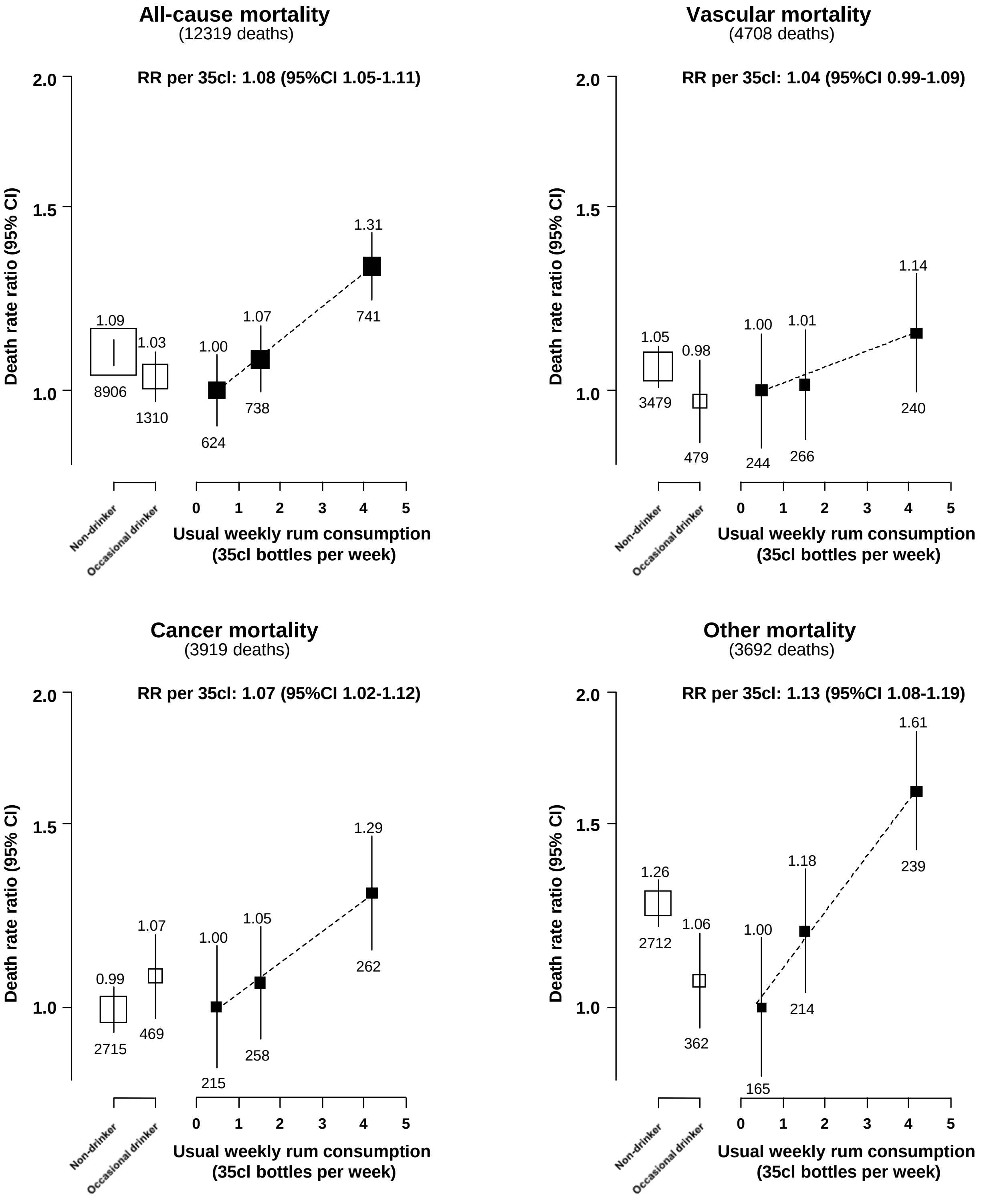

Figure S5. Alcohol consumption versus all-cause, vascular, cancer and other mortality at ages 35-79 years in Cuba, excluding participants taking anti-hypertensives

Rate ratios (RR) adjusted for age, sex, education, province, and smoking. Exclusions and conventions as per table 1, with further exclusion of those taking antihypertensive medication.

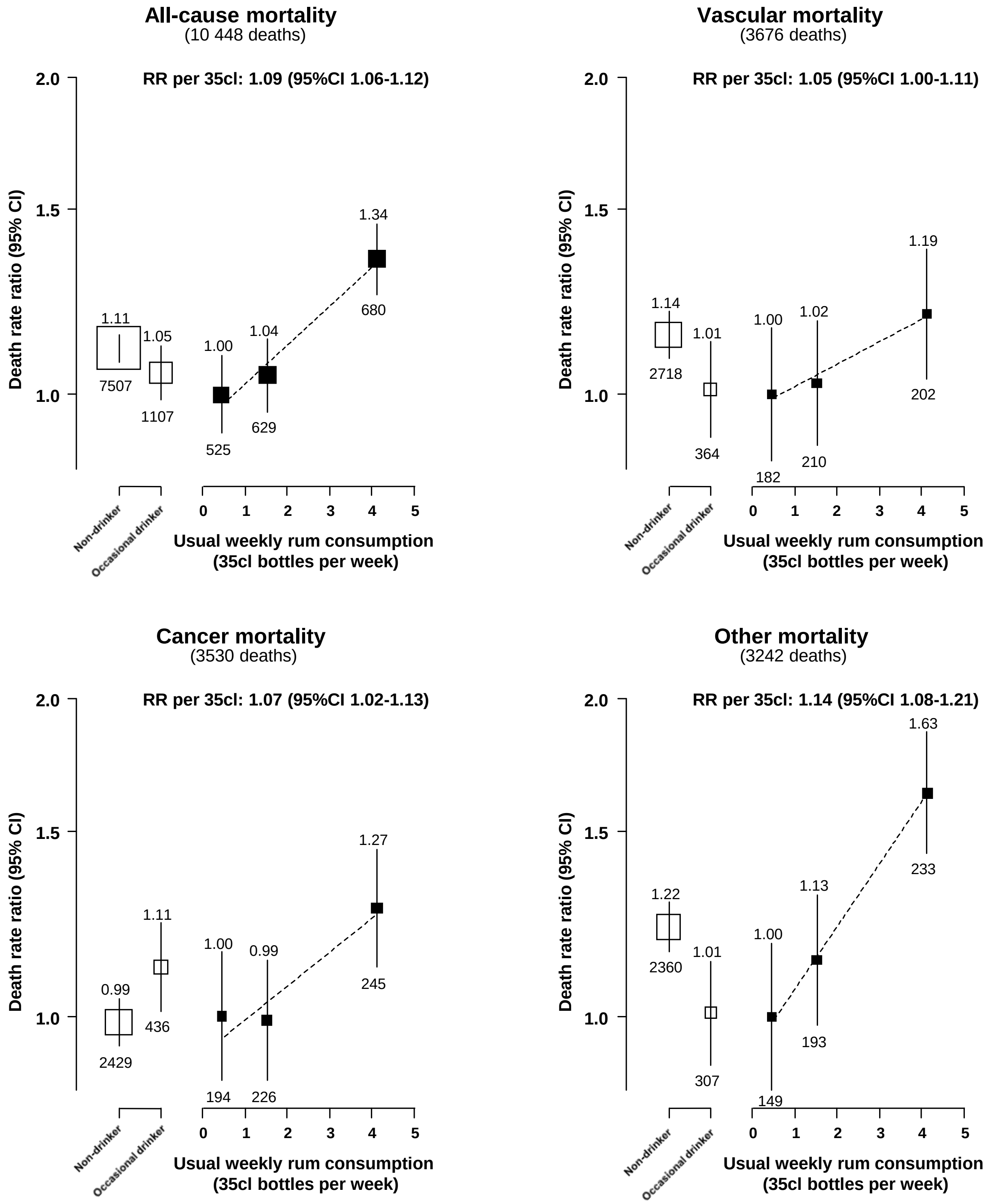

Supplement: Supplementary file 1 [file mmc1.pdf]
